# iTRAQ-Based Comparative Proteomic Analysis of *Acinetobacter baylyi* ADP1 Under DNA Damage in Relation to Different Carbon Sources

**DOI:** 10.3389/fmicb.2019.02906

**Published:** 2020-01-14

**Authors:** Bo Jiang, Yi Xing, Guanghe Li, Nana Zhang, Luning Lian, Guangdong Sun, Dayi Zhang

**Affiliations:** ^1^School of Energy and Environmental Engineering, University of Science and Technology Beijing, Beijing, China; ^2^Beijing Key Laboratory of Resource-Oriented Treatment of Industrial Pollutants, University of Science and Technology Beijing, Beijing, China; ^3^School of Environment, Tsinghua University, Beijing, China; ^4^State Key Joint Laboratory of Environmental Simulation and Pollution Control, Tsinghua University, Beijing, China

**Keywords:** *Acinetobacter baylyi* ADP1, DNA damage response, proteomics, carbon source, iTRAQ

## Abstract

DNA damage response allows microorganisms to repair or bypass DNA damage and maintain the genome integrity. It has attracted increasing attention but the underlying influential factors affecting DNA damage response are still unclear. In this work, isobaric tags for relative and absolute quantification (iTRAQ)-based proteomic analysis was used to investigate the influence of carbon sources on the translational response of *Acinetobacter baylyi* ADP1 to DNA damage. After cultivating in a nutrient-rich medium (LB) and defined media supplemented with four different carbon sources (acetate, citrate, pyruvate, and succinate), a total of 2807 proteins were identified. Among them, 84 proteins involved in stress response were significantly altered, indicating the strong influence of carbon source on the response of *A. baylyi* ADP1 to DNA damage and other stresses. As the first study on the comparative global proteomic changes in *A. baylyi* ADP1 under DNA damage across nutritional environments, our findings revealed that DNA damage response in *A. baylyi* ADP1 at the translational level is significantly altered by carbon source, providing an insight into the complex protein interactions across carbon sources and offering theoretical clues for further study to elucidate their general regulatory mechanism to adapt to different nutrient environments.

## Biological significance

*Acinetobacter baylyi* ADP1 is a well-established soil model microorganism for gene manipulation for its natural transformation ability. Its DNA damage response mechanism is not well-understood and remains unclear. A gel-free quantitative proteomic analysis (iTRAQ combined with LC/MS/MS) in this study explored the proteomic profiling of *A. baylyi* ADP1 with mitomycin C-induced DNA damage and cultivated with different carbon sources. This work illustrates a comprehensive picture of the dynamic changes in proteomics of *A. baylyi* ADP1 response to DNA damage stress, providing a deeper and broader understanding of the carbon source-dependent DNA damage response. Proteomics has great potential as an advanced technique for analyzing stress responses of microbes at the translational level.

## Introduction

A variety of sources can induce stress to bacterial cells, e.g., radiation (Sankaranarayanan, 1996), heat (Roncarati and Scarlato, 2017), salt (Egamberdieva et al., 2017), chemical mutagens (Abraham et al., 2006), carbon starvation (Handtke et al., 2018), and metabolites (Vainio et al., 1981; Kulling et al., 2002). To survive and thrive in these extreme conditions, microbes have a repertoire of genes that can be activated or silenced responding to stress (van der Veen and Abee, 2011). Although numerous works have attempted to unravel the changes in gene expression and mRNA transcription under stress response (Zhou et al., 2016; Kotrade et al., 2019; Qiao et al., 2019; Szabo et al., 2019), evidence has shown poor correlations between transcriptional and protein levels (Pascal et al., 2008). Accordingly, recent studies have employed proteomic analysis to investigate microbial global response to environmental stresses, e.g., antibiotic stress (Mathieu et al., 2016; Xiong et al., 2017), cold and light stress (Liu et al., 2018), oxidative stress (van Herwijnen et al., 2003), drying stress (Schott et al., 2017), thermal stress (Delgado-Baquerizo et al., 2018), and nitrate and phosphate depletion (Lin et al., 2017).

As a global response to DNA damage, the inducible DNA repair network was firstly found in *Escherichia coli* (Radman, 1975), named SOS response. SOS response network is highly conserved and widely present in bacteria, allowing them to repair or bypass DNA damage and maintain genome integrity (Friedman et al., 2005; Chaudhary et al., 2011). In addition, SOS response network is a key driving force for the evolution of bacterial genomes (Michel et al., 2004; Stavans, 2006), which is also linked to virulence and stress-induced mutagenesis (Sanchez-Alberola et al., 2012). Till now, SOS response network in *E. coli* is well studied that a LexA/RecA-dependent SOS response system consists of more than 40 enzymes performing diverse functions responding to DNA damage, e.g., homologous recombination, nucleotide excision repair (NER mechanism), and translesion DNA replication (Khan et al., 2001; Friedman et al., 2005; Meng and Zhu, 2011). Although other bacterial strains have similar DNA damage response mechanisms in comparison with that in *E. coli* (Rauch et al., 1996; Booth et al., 2001; Brezna et al., 2003; DeBruyn et al., 2012), different functional enzymes are found across species. For instance, a soil model microorganism *Acinetobacter baylyi* ADP1 has UmuDAb protein possessing a post-translational and LexA-like cleavage after DNA damage (Hare et al., 2012). Another recent study found a PafB/PafC-regulated DNA damage response network in *Mycobacteria* and other *Actinobacteria* strains (Olivencia et al., 2017). In addition, proteomics analysis has identified diverse proteins under DNA damage stress. For example, the proteomic response of *Cryptococcus podzolicus* Y3 to citrinin suggested that the up- and down-regulated proteins were associated with structural maintenance of chromosomes (DNA double-strand break repair Rad50 ATPase, etc.), cell apoptosis (cytochrome C), detoxification and energy metabolism (Glyco-syltransferase and malate dehydrogenase), and oxidative stress response (superoxide dismutase [Cu-Zn] and cysteine peroxiredoxin) (Wang et al., 2019).

DNA damage response, at the translational level, is reported to correlate with many environmental variables, e.g., pH (Wu et al., 2018), nutrient fluctuations (Lin et al., 2017), and carbon sources (Seo et al., 2013; Jiang et al., 2015). Among them, carbon source is essential for heterotrophic microorganisms and can influence protein profiles involved in carbohydrate transport and metabolism, energy metabolism, nucleotide metabolism, stress response, and protein biosynthesis (Halimaa et al., 2013; Moreno and Rojo, 2013; Siragusa et al., 2014; Liu et al., 2015; Mahar et al., 2016; Li et al., 2019).

*Acinetobacter baylyi* ADP1 is a well-established soil microorganism for gene manipulation owing to its natural transformation ability (Barbe et al., 2004) and tolerance to environmental stress (Al-Anizi et al., 2014). Many studies have constructed various bioreporters with *A. baylyi* ADP1 as hosts for genotoxicity assessment of environmental samples (Song et al., 2009, 2014; Zhang et al., 2013; Jiang et al., 2014, 2017; Jia et al., 2016). A previous study has documented that the DNA damage response in *A. baylyi* ADP1 was dependent on carbon source at both transcriptional and translational levels (Jiang et al., 2015). Such behavior might affect the performance of *A. baylyi* ADP1 bioreporters in environmental monitoring, but there is lack of detailed mechanisms of protein profiles change in response to genotoxins cultivated with different carbon sources. Therefore, a deeper insight into the carbon-dependent protein profiles gains our knowledge on the key proteins and processes involved in DNA damage response, benefiting the understanding of DNA damage response network in *A. baylyi* ADP1 and optimization of *A. baylyi* hosted bioreporters.

In this work, we studied the influence of carbon sources on proteomic profiles of *A. baylyi* ADP1 in response to DNA damage induced by mitomycin C. Besides a nutrient-rich medium Luria-Bertani Broth (LB), *A. baylyi* ADP1 cells were also cultivated in defined media with acetate, citrate, pyruvate, and succinate as sole carbon source, to explore the difference in proteomic profiles *via* isobaric tags for relative and absolute quantification (iTRAQ) coupled with liquid chromatography coupled with mass spectrometry (LC/MS/MS). Proteins associated with different functions and biological processes were compared, and up- or down-regulated proteins related to energy production and stress response were particularly addressed. A protein–protein network was built to elucidate the protein interactions. This work provides the first comprehensive discussion on DNA damage response of *A. baylyi* ADP1 under different carbon source conditions, aiming at a better understanding of DNA damage stress response and to improve the performance of *A. baylyi* ADP1 hosted bioreporters.

## Materials and Methods

### Chemicals and Bacterial Strains

Unless specifically stated, all the reagents used in this study were purchased from Sigma-Aldrich (St. Louis, MO, United States) and of analytical grade. In this work, *A. baylyi* ADP1 was the model strain to evaluate the carbon-dependent proteome profiles in response to DNA damage. Nutrient-rich medium was LB medium, which consists of 5 g NaCl, 5 g yeast extract, and 10 g peptone in 1.0 L of sterilized water (pH adjusted to 7.0). Nutrient-deficient medium was prepared from minimal medium (MM), which consists of 0.5 g KH_2_PO_4_, 0.5 g NaCl, 0.1 g CaCl_2_, 0.2 g MgSO_4_⋅7H_2_O, 0.5 g FeSO_4_⋅7H_2_O, 0.5 g MnSO_4_, and 1.5 g (NH_4_)_2_SO_4_ in 1.0 L of sterilized water (Zhang et al., 2015). MM was then supplemented with different carbon sources (Zhang et al., 2011), and the final concentration of potassium acetate, sodium citrate, sodium pyruvate, and sodium succinate was 30 mM, designated as MMA, MMC, MMP, and MMS, respectively.

### DNA Damage Treatments

Mitomycin C is a bio-reductive alkylating agent causing DNA cross-linking and genotoxic effects (Abraham et al., 2006), viewed as a classic approach inducing DNA damage and used in this work to investigate the proteomic changes and global SOS response network in response to DNA damage in *A. baylyi* ADP1 among different carbon sources. In principle, *A. baylyi* ADP1 was firstly cultivated in nutrient-rich medium (LB) and then exposed to DNA damage induced by mitomycin C in both nutrient-rich medium (LB) and nutrient-deficient media (MMA, MMC, MMP, and MMS), respectively. To be more precise, *A. baylyi* ADP1 cells were inoculated in LB overnight at 30°C and harvested by centrifugation at 4000 × *g* for 10 min. After resuspended in the same volume of sterile deionized water, 1 ml of cell suspension was supplemented with 9 ml of fresh medium (LB, MMA, MMC, MMP, and MMS; carbon source final concentration, 27 mM, which falls in the range of 10–40 mM as conventional protocols for cell cultivation) (Selifonova et al., 1993; Zhang et al., 2012, 2013; Jebasingh et al., 2013; Borirak et al., 2015; Jia et al., 2016; Schmidt et al., 2016) and cultivated at 30°C until the early exponential phase when optical density at 600 nm (OD_600_) was 0.10. The suspensions were further added with mitomycin C to the final concentration of 1 μM to induce DNA damage. After 3-h exposure, cells were harvested by centrifugation at 6000 × *g* for 15 min at 4°C for further analysis. Although some previous studies have reported the application of 3 μM mitomycin C to induce *A. baylyi* cells, the concentration of mitomycin C used in the present study was set as 1 μM, because our previous work has reported a linear detection range of 0.1 nM to 1 μM for ADPWH_recA postexposure to mitomycin C (Jiang et al., 2017). Accordingly, mitomycin C concentration at 1 μM could induce the highest bioluminescent response of bioreporter cells, whereas higher concentration could significantly inhibit cell growth and might affect proteomic profiles (Song et al., 2009).

### Cell Lysis, Protein Extraction, and Protein Digestion

Cell pellets were suspended in a protein extraction buffer (urea [6.0 M], ethylenediamine tetra-acetic acid [EDTA, 0.5 mM], sodium dodecyl sulfate [SDS, 2%, w/v], and protease inhibitors cocktail mixture [Roche] in NH_4_HCO_3_ [100 mM] lysis buffer) (Borirak et al., 2015), followed by vortex and sonication (Branson Sonifier 450 D, United States). Sonication was set at 300 W in a pulse mode (10 s/10 s) for 20 min at ice bath until the cell suspension became clear. Protein concentration was measured by the Bradford assay (Pierce^TM^ Coomassie [Bradford] Protein Assay Kit, Thermo Scientific, United Kingdom).

Subsequently, around 100 μg of proteins was subjected to trypsin digestion as previously reported (Pereira et al., 2011). Briefly, 2 μl of Reducing Reagent (supplied in B-PER^TM^ Complete Bacterial Protein Extraction Reagent, Thermo Scientific, United Kingdom) was added in protein suspension and incubated at 60°C for 1 h. Another 1 μl of Cysteine-Blocking Reagent was added, kept at room temperature for 10 min, and centrifuged for 20 min to discard the supernatant. Subsequently, 100 μl of Dissolution Buffer (supplied by iTRAQ^®^ Reagent-8PLEX Multiplex Kit, Sigma-Aldrich, United States) was added and transferred to a new collection tube. Trypsin was added, incubated at 37°C overnight, and centrifuged for 20 min twice to collect the digested peptides. The quantity and quality of proteins and trypsin digestion efficiency were analyzed by 10% acrylamide gels.

### iTRAQ Labeling, LC/MS/MS Analysis, and Data Processing

The analysis of proteomics was performed using iTRAQ technology by Beijing Proteome Research Centre (Supplementary Figure S1). Briefly, 100 μg of digested peptides was labeled with amine-reactive isobaric tags (supplied in iTRAQ^®^ Reagent-8PLEX Multiplex Kit, Sigma-Aldrich, United States), incubated at room temperature for 2 h, and stopped by 100 μl of water. Qualification and quantification of peptides were performed by LC/MS/MS. LC fractionation was performed in binary gradient buffers (buffer A and B). The labeled samples were resuspended in buffer A (10 mM NH_4_HCO_3_, 80% acetonitrile, pH 3.0). The binary gradient began with 5% B (10 mM NH_4_HCO_3_, 5% acetonitrile, pH 4.0) for 5 min, a linear ramp from 8% to 32% B for 59 min, an extended ramp from 32% to 95% B for 4 min, a further isocratic wash with 95% B for 5 min, and column re-equilibration with 5% B for 2 min. The injection volume was 100 μl and the chromatographic flow rate was constantly at 0.7 ml/min.

Each fragmented peptide tag produced distinct signature ions, which were detected by mass spectrometry (TripleTOF^TM^ ABI-5600, Applied Biosystems, Wilmington, DE, United States) and distinguished by *m/z* value (Zilberstein, 2015). Data acquisition was in the positive ion mode with an accumulation time of 1 s. The selected mass detector ranged from 400 to 1250 *m/z*, and the precursor ion scan was performed within a range of 330–2000 *m/z*.

The original data were processed by ProteinPilot^TM^ Software 4.5 (Applied Biosystems, Wilmington, DE, United States), using a database comprising all *Acinetobacter* protein sequences obtained from National Center for Biotechnology Information (NCBI) database. Acceptance threshold for peptide identification was length ≥ 6, *z*-score ≥ 5 and *p*-value ≤ 0.05. A decoy database created by reversing the sequences was used to calculate the false discovery rate (FDR), and proteins identified with at least two peptides and satisfying a 5% FDR were kept for quantitative analysis. The abundance of each peptide in LB treatment was set as the reference, and the relative abundance was calculated as the ratio of the abundance of each peptide in other treatments (MMA, MMC, MMP, and MMS) to that in LB.

### Data Analysis

Based on the well-accepted method to classify the up- and down-regulation using *p*-value in *t*-test (Lin et al., 2017; Fountain et al., 2018), peptides with relative abundance < 0.5 (*p* < 0.01), 0.5–0.83 (*p* < 0.05), 1.2–2.0 (*p* < 0.05), and >2.0 (*p* < 0.01) were designated in the present study to be significantly up-regulated or down-regulated. They were further classified according to the Gene Ontology (GO) function and GO biological process, *via* the Universal Protein Resource Database^1^ (O’Donovan and Apweiler, 2011; Lai et al., 2017) and PSORTdb 3.0.^2^ Network analysis of differentially expressed proteins was performed by String Software (V11.0): Protein–Protein Interaction Networks^3^. Statistical analysis was carried out by SPSS (version 21.0) to evaluate the correlations of the relative abundance of all the identified peptides between different carbon source treatments.

## Results

### Annotation of Protein Functions and Biological Processes

A total of 2807 proteins with function prediction were identified in all the treatments (Supplementary Figure S2). Analyzed by PSORTdb database and GO, they were categorized into 26 functions, and the most abundant ones included transferase (328 proteins, 11.7%), nucleic acid-binding protein (243 proteins, 8.6%), oxidoreductase (282 proteins, 10.0%), hydrolase (262 proteins, 9.3%), transporter (106 proteins, 3.8%), and lyase (93 proteins, 3.3%) (Supplementary Figure S2A). As for biological processes, most identified proteins were associated with biosynthesis process (257 proteins, 22.5%), metabolite process (219 proteins, 19.2%), transport (149 proteins, 13.1%), amino acid biosynthesis (91 proteins, 7.9%), regulator (73 proteins, 6.4%), and stress response (70 proteins, 6.1%) (Supplementary Figure S2B).

### Comparison of Protein Functions and Biological Processes in Different Carbon Sources

To investigate the distinct protein profiles in rich (LB) and defined media (MMA, MMC, MMP, and MMS), the numbers of up- or down-regulated proteins in MMA, MMC, MMP, and MMS treatments were calculated and listed in Table 1. Of all the 2807 identified proteins, the percentage of proteins with significant changes in MMA, MMC, MMP, and MMS was 52.1, 57.9, 51.1, and 48.2%, respectively. It indicated that the relative abundance of approximately half of the proteins was affected by carbon sources. Among the four defined media, the changes in peptide levels in MMC varied most greatly comparing to LB, with 29.5% proteins down-regulated and 28.3% proteins up-regulated, and MMS had 23.4% of proteins down-regulated and 24.7% of proteins up-regulated. However, the up- or down-regulated proteins are different across carbon sources, suggesting that the change of protein profiles is dependent on carbon sources.

**TABLE 1:** Number of proteins up- or down- regulated in defined media (MMA, MMC, MMP, and MMS) in comparison with LB.

**Treatment**	**MMA**	**MMC**	**MMP**	**MMS**
Significantly down-regulated^1^	149	210	134	113
Down-regulated to some extent^2^	595	620	545	545
Unchanged^3^	1345	1183	1373	1455
Up-regulated to some extent^4^	540	562	551	541
Significantly up-regulated^5^	178	232	204	153

*^1^The ratio of protein expression level in defined medium to that in LB was lower than 0.5 (*p* < 0.01). ^2^The ratio of protein expression level in defined medium to that in LB was between 0.5 and 0.83 (*p* < 0.05). ^3^The ratio of protein expression level in defined medium to that in LB was between 0.83 and 1.2 (*p* > 0.05). ^4^The ratio of protein expression level in defined medium to that in LB was between 1.2 and 2.0 (*p* < 0.05). ^5^The ratio of protein expression level in defined medium to that in LB was higher than 2.0 (*p* < 0.01).*

**FIGURE 1:**
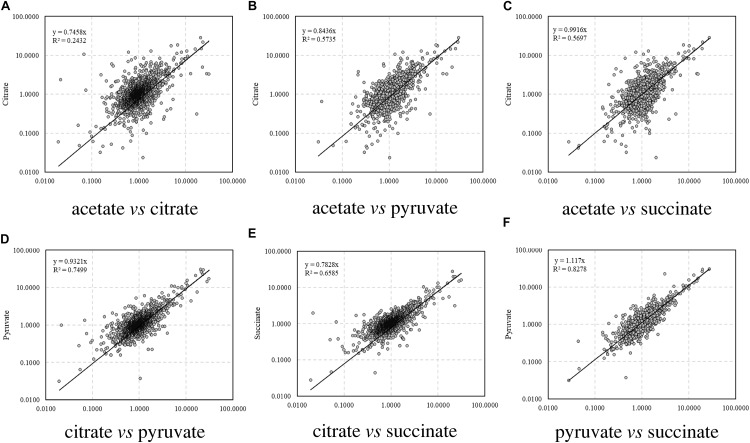
Correlation of relative abundance of proteins among carbon sources. **(A)** Acetate vs. citrate; **(B)** acetate vs. pyruvate; **(C)** acetate vs. succinate; **(D)** citrate vs. pyruvate; **(E)** citrate vs. succinate; **(F)** pyruvate vs. succinate.

The correlations of the relative abundance of all the identified peptides between different carbon source treatments are illustrated in Figure 1. The correlation coefficient (*R*), which is a statistical measure representing the strength of the relationship ranging from −1.0 and 1.0, is used to evaluate the similarity of protein profiles and a higher *R* value indicates a stronger positive relationship. Among the four treatments, the relative abundance of proteins in citrate treatment differed remarkably from that in acetate (*R*^2^ = 0.2432, Figure 1A), whereas the proteomic profiles were similar between acetate and pyruvate treatments (*R*^2^ = 0.5735, Figure 1B), and acetate and succinate treatments (*R*^2^ = 0.5697, Figure 1C). The proteomic profiles in pyruvate and succinate treatments showed the highest consistency (*R*^2^ = 0.8278, Figure 1F).

**FIGURE 2:**
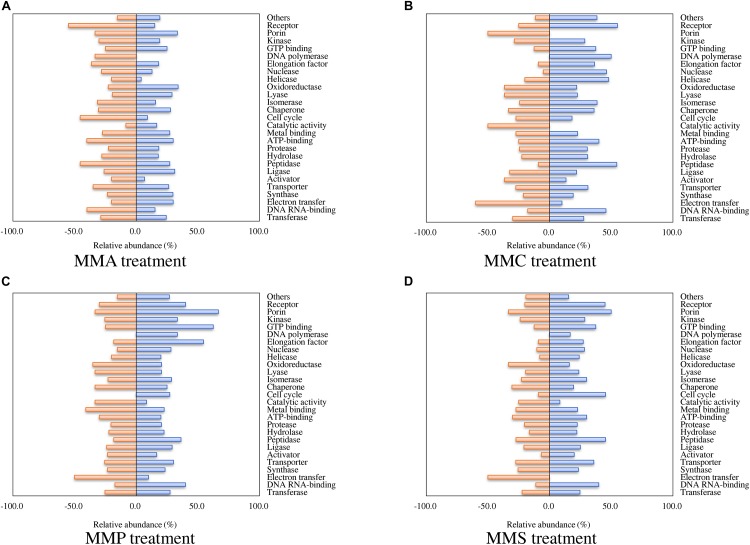
Percentage of proteins with significant fold changes in each carbon source treatment compared to LB medium. **(A)** MMA; **(B)** MMC; **(C)** MMP; **(D)** MMS. Proteins are classified by functions, and the categories with numbers less than 10 are summed up as “others.”

According to the classification of protein function, the percentages of proteins with significant fold changes in each carbon source treatment are illustrated in Figure 2. Proteins with each function had their unique profiles among carbon sources. The relative abundance of some proteins showed similar tendency in all treatments, e.g., transferase (24.4–27.7% up-regulated and 22.3–29.9% down-regulated), synthase (19.1–29.8% up-regulated and 21.3–25.5% down-regulated), and transporter (26.4–35.8% up-regulated and 25.5–34.9% down-regulated). The regulation of some other proteins varied significantly across treatments. For instance, 55% of receptor proteins were down-regulated in MMA treatment, whereas only 25, 20, and 30% of them were down-regulated in MMC, MMP, and MMS treatments, respectively. Some proteins even demonstrated entirely opposite patterns. Among them, DNA polymerase was up-regulated in MMC, MMP, and MMS treatments, but down-regulated in MMA treatment. Interestingly, the percentage of up-regulated proteins related to DNA replication was remarkably higher in MMC than that in other treatments, e.g., DNA polymerase (50.0% up-regulated, for DNA synthesis from a DNA template), helicase (48.0% up-regulated, for the unwinding of double-stranded helical structure of nucleic acids), nuclease (46.2% up-regulated, for hydrolysis of nucleic acids), as well as peptidase (54.5% up-regulated, for the breakdown of protein peptides).

**FIGURE 3:**
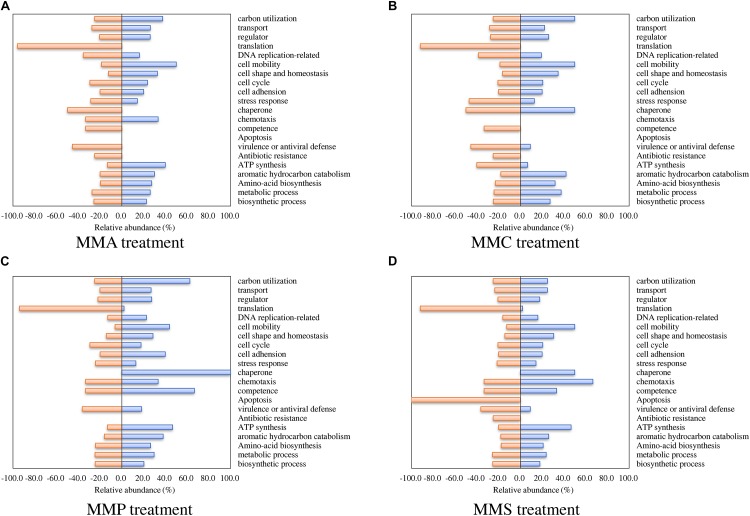
Percentage of proteins with significant fold changes in each carbon source treatment compared to LB medium. **(A)** MMA; **(B)** MMC; **(C)** MMP; **(D)** MMS. Proteins are classified by biological process category.

Regarding biological processes, the percentages of up- and down-regulated proteins across different carbon source treatments are shown in Figure 3. Among them, proteins related to biosynthetic process, metabolic process, amino acid biosynthesis, and aromatic hydrocarbon catabolism showed similar patterns in all treatments. A significant repression effect on translation-related proteins was found in all treatments, in which 93.5% ± 1.9% of translation-related proteins were down-regulated. Proteins involved in ATP synthesis behaved differently between MMC (6.7% up-regulated and 40.0% down-regulated), MMA (40.0% up-regulated and 13.3% down-regulated), MMP (46.7% up-regulated and 13.3% down-regulated), and MMS (46.7% up-regulated and 20.0% down-regulated). Additionally, proteins associated with response in adverse conditions were mostly down-regulated, including proteins for stress response (30.4% ± 11.6%), virulence or antiviral defense (40.9% ± 5.3%), and antibiotic resistance (18.8% ± 12.5%).

### Different Protein Profiles Between Nutrient-Deficient and Nutrient-Rich Medium

Of the proteins with significant fold changes in comparison with LB, 174 and 210 proteins exhibited the same up- or down-regulation patterns across different carbon source treatments, respectively. Among them, 140 and 166 proteins were validated or had putative roles in metabolic processes (Table 2). Proteins associated with four types of functions were most significantly affected, including transferase (34 up-regulated and 27 down-regulated), hydrolase (11 up-regulated and 16 down-regulated), oxidoreductase (37 up-regulated and 12 down-regulated), and DNA/RNA-binding proteins (7 up-regulated and 60 down-regulated).

**TABLE 2:** Up- and down-regulated proteins involved in each molecular functional category.

**Protein**	**Gene name**	**ACIAD No.**	**MMA:LB**	**MMC:LB**	**MMP:LB**	**MMS:LB**
**Up-regulated**						
**Transferase**						
Acetyltransferase	*argA*	ACIAD0039	1.61	1.53	1.74	1.49
2-isopropylmalate synthase	*leuA*	ACIAD0530	6.49	11.07	6.55	6.08
Phosphate acetyltransferase	*pta*	ACIAD0540	1.82	3.87	3.16	1.75
Acetate kinase (propionate kinase)	*ack*	ACIAD0541	1.84	4.02	3.53	2.07
Branched-chain amino acid aminotransferase	*ilvE*	ACIAD0597	2.54	1.5	1.31	1.2
Glutamate synthase subunit alpha	*argJ*	ACIAD0650	5.5	7.52	7.8	7.59
Glutamate synthase subunit beta	*argJ*	ACIAD0650	5.75	8.47	10.67	8.87
UDP-*N*-acetylglucosamine 1-carboxyvinyltransferase	*murA*	ACIAD0660	1.33	1.25	1.32	1.29
*O*-methyltransferase protein	*bioC*	ACIAD0858	2.4	1.96	1.77	1.32
Acyl carrier protein	*acpP*	ACIAD0872	3.87	3.87	4.21	5.11
Glycerol kinase	*glpK*	ACIAD0930	2.33	3.63	3.53	1.39
Dihydrolipoamide dehydrogenase (E3 component of pyruvate and 2-oxoglutarate dehydrogenase complexes)	*acoC*	ACIAD1019	2.86	5.5	3.77	3.8
B12-dependent methionine synthase	*metH*	ACIAD1045	1.85	1.46	1.66	1.26
Sulfate adenylyltransferase subunit 2	*cysD*	ACIAD1072	1.61	2.09	1.94	2.11
Aspartate carbamoyltransferase catalytic subunit	*pyrB*	ACIAD1270	4.53	4.83	4.7	2.68
Aspartate carbamoyltransferase	*pyrB*	ACIAD1270	2.94	2.94	2.44	2.19
NADH oxidase	*putA*	ACIAD1646	2.36	2.23	1.74	1.94
3-oxoadipate CoA-transferase	*pcaJ*	ACIAD1705	1.53	1.29	1.49	1.41
3-oxoadipate CoA-transferase subunit A	*pcaI*	ACIAD1705	2.47	3.7	2.56	1.56
Beta-ketoadipyl CoA thiolase	*pcaF*	ACIAD1706	1.58	2.68	1.91	1.28
Phospho-2-dehydro-3-deoxyheptonate aldolase	*aro*	ACIAD1878	10.96	9.38	11.07	7.66
Phosphoglycerate kinase	*pgk*	ACIAD1927	1.94	2.05	4.17	1.91
Aminotransferase AlaT	*AlaT*	ACIAD2087	2.33	2.03	1.58	1.64
Malate synthase G	*glcB*	ACIAD2335	4.66	2.94	4.66	2
Acetyl-CoA acetyltransferase	*atoB*	ACIAD2516	1.47	3.94	1.58	1.28
Sulfate ABC transporter periplasmic substrate-binding protein	*cysP*	ACIAD2591	5.4	2.75	4.74	4.29
Acyl-CoA transferase/carnitine dehydratase	−	ACIAD2821	1.87	6.03	2.38	1.51
Acetolactate synthase 3 catalytic subunit	*ilvH*	ACIAD3103	6.37	5.86	3.73	4.53
Acetolactate synthase 3 regulatory subunit	*ilvH*	ACIAD3103	3.87	2.88	2.33	1.96
Homocysteine synthase	*metY*	ACIAD3382	2.17	4.74	3.22	2.65
Dihydrolipoamide acetyltransferase	*aceF*	ACIAD3506	2.38	6.31	4.13	3.53
UDP-*N*-acetylmuramate–L-alanine ligase	*murC*	ACIAD3516	1.47	1.84	1.89	2.11
5-methyltetrahydropteroyltriglutamate–homocysteine methyltransferase	*metE*	ACIAD3523	28.31	3.37	14.19	14.86
NADH dehydrogenase II	*ndh*	ACIAD3633	1.84	2.25	4.33	2.75
**DNA/RNA-binding**						
AraC family transcriptional regulator	*ntrC*	ACIAD0194	1.27	1.67	1.54	1.49
Curved DNA-binding protein	*cbpA*	ACIAD0406	1.54	1.96	1.96	1.5
Magnesium and cobalt efflux protein	*corC*	ACIAD0416	1.87	1.37	1.25	1.6
Transcriptional regulator	*baeR*	ACIAD0627	5.5	3.84	3.7	5.45
AraC family transcriptional regulator	*glnG*	ACIAD1368	1.26	3.94	2.73	1.26
Holliday junction DNA helicase RuvB	*ruvB*	ACIAD2615	1.51	1.29	1.32	1.32
Two-component regulatory system response regulator	*ompR*	ACIAD3388	1.92	2	1.82	2.13
**Electron transfer**						
Tryptophan synthase beta chain	*trpB*	ACIAD0636	1.46	2.01	1.27	1.31
Ferredoxin 1	*fpr*	ACIAD2244	1.54	1.67	2.01	1.91
Electron transfer flavoprotein beta-subunit	*etfB*	ACIAD2655	1.41	1.64	1.87	2.03
**Synthase**						
Cysteinyl-tRNA synthetase	*cysS*	ACIAD1481	1.42	1.43	1.69	1.42
Citrate synthase	*gltA*	ACIAD2886	3.53	2.09	4.61	1.79
**Transporter**						
Response regulator	*gacA*	ACIAD0260	1.29	1.45	1.21	1.54
Glutamate/aspartate ABC transporter substrate-binding protein	*gltK*	ACIAD2058	0.7	7.52	1.89	1.36
Outer membrane protein (ComQ)	*comQ*	ACIAD3355	4.17	3.87	3.31	3.98
**Activator**						
Transcriptional regulator	*qseB*	ACIAD2961	5.5	3.84	3.7	5.45
**Ligase**						
Cysteinyl-tRNA synthetase	*cysS*	ACIAD1481	1.42	1.43	1.69	1.42
Medium-chain-fatty-acid-CoA ligase	*alkK*	ACIAD1818	1.39	5.6	3.16	1.31
Protein kinase	*pyrG*	ACIAD2003	2.15	1.36	1.89	1.58
Glutamine synthetase	*glnA*	ACIAD2458	2.25	2.86	2.63	2.56
Carbamoyl phosphate synthase, large subunit	*carB*	ACIAD2861	3.5	3.37	1.66	1.84
Acetyl-CoA synthetase	*acs*	ACIAD3463	2.23	5.86	3.98	2.96
**Peptidase**						
Aminopeptidase P	*pepP*	ACIAD1126	1.82	1.58	1.87	1.49
**Hydrolase**						
Thiol: disulfide interchange protein (DsbC-like)	*dsbA*	ACIAD0045	2.29	3.22	3.4	2.75
Bifunctional phosphoribosyl-AMP cyclohydrolase/phosphoribosyl-ATP pyrophosphatase	*hisIE*	ACIAD0380	1.54	1.39	1.29	1.29
SlyX protein	*slyX*	ACIAD0488	1.5	1.71	1.67	1.24
Hydrolase	–	ACIAD0886	3.8	2.96	2.81	2.96
Multidrug resistance secretion protein	*emrA*	ACIAD0926	1.46	1.22	1.63	1.24
ATP-binding protease component	*clpA*	ACIAD1363	1.49	1.91	1.33	1.57
Salicylate esterase	*SalE*	ACIAD1426	2.65	3.19	2	2.11
3-oxoadipate enol-lactonase 2	*catD*	ACIAD1451	1.54	1.69	1.72	1.37
Fructose-1,6-bisphosphate aldolase	*fda*	ACIAD1925	2.23	1.82	1.61	1.38
Copper transporting ATPase	*copA*	ACIAD2400	1.49	2.19	2.05	2
Toluene tolerance efflux ABC transporter periplasmic substrate-binding protein	*ttg2C*	ACIAD3242	1.33	1.72	1.32	1.61
**Protease**						
SlyX protein	*slyX*	ACIAD0488	1.5	1.71	1.67	1.24
Aminopeptidase P	*pepP*	ACIAD1126	1.82	1.58	1.87	1.49
ATP-binding protease component	*clpA*	ACIAD1363	1.49	1.91	1.33	1.57
**ATP-binding**						
Twitching motility protein	*pilG*	ACIAD0786	3.4	3.66	5.55	3.94
Twitching motility protein	*pilH*	ACIAD0787	2.31	2.94	2.99	2.58
Twitching motility protein	*pilI*	ACIAD0788	1.26	1.41	1.26	1.2
**Metal binding**						
Bacterioferritin	*bfrA*	ACIAD0852	2.03	2.38	2.38	2.56
Bacterioferritin	*bfrB*	ACIAD3330	2.65	3.37	2.86	2.7
**Chaperone**						
Fimbrial chaperone protein	–	ACIAD0388	2.61	2.05	2.49	2.07
Fimbrial protein	–	ACIAD0390	2.65	2.19	2.7	2.47
ATP-binding protease component	*clpA*	ACIAD1363	1.49	1.91	1.33	1.57
Lipase chaperone	*lifO*	ACIAD3308	1.39	3.19	2.25	1.54
**Isomerase**						
Peptidyl-prolyl *cis*-*trans* isomerase	*fklB*	ACIAD0065	1.33	2.05	1.85	1.56
UDP-*N*-acetylglucosamine 1-carboxyvinyltransferase	*murA*	ACIAD0660	1.33	1.25	1.32	1.29
Peptidylprolyl isomerase	*ppiD*	ACIAD1409	2.33	2.38	1.5	1.69
Peptidyl-prolyl *cis-trans* isomerase precursor (PPIase)	*surA*	ACIAD2372	1.74	1.29	1.98	1.21
**Lyase**						
Threonine synthase	*thrC*	ACIAD0263	2.47	1.71	1.38	1.92
Thiamine biosynthesis protein ThiC	*thiC*	ACIAD0276	20.7	28.31	30.2	27.54
Isopropylmalate isomerase large subunit	*leuC*	ACIAD0463	23.33	21.68	29.38	19.77
Isopropylmalate isomerase small subunit	*leuD*	ACIAD0466	16.14	14.32	20.7	12.82
Fumarate hydratase	*fumA*	ACIAD0538	5.7	4.33	3.47	3.6
Isocitrate lyase	*aceA*	ACIAD1084	22.91	4.88	22.28	3.08
Dihydroxy-acid dehydratase 1	*ilvD1*	ACIAD1266	3.98	3.94	2.96	2.42
4-carboxymuconolactone decarboxylase	*pcaC*	ACIAD1710	2.49	1.94	2.05	2.13
Catabolic 3-dehydroquinate dehydratase	*aroD*	ACIAD1713	2.4	4.97	2.75	2.33
Aconitate hydratase	*acnA*	ACIAD3090	1.94	1.51	1.56	1.91
Dihydroxy-acid dehydratase 2	*ilvD2*	ACIAD3636	3.08	6.55	7.24	4.13
**Oxidoreductase**						
Flavoprotein monooxygenase acting on aromatic compound	–	ACIAD3540	1.41	1.43	1.98	2.01
NADH-flavin reductase	–	ACIAD3290	1.69	1.58	1.21	2.01
Magnesium and cobalt efflux protein	*corC*	ACIAD0416	1.87	1.37	1.25	1.6
3-isopropylmalate dehydrogenase	*leuB*	ACIAD0469	10.67	12.82	15.56	10
Aldehyde dehydrogenase	*calB*	ACIAD0503	8.17	11.8	9.38	8.39
Peptide methionine sulfoxide reductase	*msrA*	ACIAD0510	1.61	2.65	2.31	2.15
Dihydrofolate reductase	*folA*	ACIAD0514	1.49	1.22	1.47	1.21
Acyl-CoA dehydrogenase	*acdB*	ACIAD0624	1.64	1.94	1.46	2.23
NADH-quinone oxidoreductase subunit K	*nuoK*	ACIAD0740	1.41	1.56	1.46	1.61
Thioredoxin reductase 1	*trxB*	ACIAD0890	2.13	1.91	2.09	1.58
Acetoin:2,6-dichlorophenolindophenol oxidoreductase alpha subunit	*acoA*	ACIAD1017	2.78	5.3	3.44	2.44
Diacetyl reductase	*budC*	ACIAD1022	1.82	6.79	2.99	1.8
Isocitrate dehydrogenase	*icd*	ACIAD1190	2.68	3.91	2.56	3.05
Aryl-alcohol dehydrogenase	*areB*	ACIAD1429	2.81	8.24	2.47	3.31
Linalool 8-monooxygenase	*linC*	ACIAD1575	1.25	1.27	1.31	1.36
Methylmalonate-semialdehyde dehydrogenase	*mmsA*	ACIAD1604	2.33	5.7	3.19	3.44
3-hydroxyisobutyrate dehydrogenase	*mmsB*	ACIAD1605	2.44	3.44	2.7	1.47
NADH oxidase	*putA*	ACIAD1646	2.36	2.23	1.74	1.94
Protocatechuate 3,4-dioxygenase subunit beta (3,4-PCD)	*pcaH*	ACIAD1711	1.96	2.25	1.75	1.56
Protocatechuate 3,4-dioxygenase subunit alpha (3,4-PCD)	*pcaG*	ACIAD1712	3.31	3.19	2.65	3.1
Hydroxybenzaldehyde dehydrogenase	*hcaB*	ACIAD1725	1.69	3.53	1.45	1.94
Dihydropteridine reductase	*nfnB*	ACIAD1923	1.69	1.25	1.82	1.41
Glutathione peroxidase	*gpo*	ACIAD2085	1.2	2.88	2.49	2.01
Ferredoxin-NADP^+^ reductase	*fpr*	ACIAD2244	2.03	1.89	1.98	1.96
Malic enzyme	–	ACIAD2287	2.56	4.41	3.44	2.61
Signal peptide	*lepB*	ACIAD2583	2.63	1.71	2.11	1.77
Glutamate dehydrogenase	*gdh*	ACIAD2680	3.94	5.65	4.66	3.34
Succinate dehydrogenase flavoprotein subunit	*sdhA*	ACIAD2880	3.28	1.82	1.85	2.96
Glucose dehydrogenase	*gcd*	ACIAD2983	1.43	1.2	1.41	1.22
Pyridine nucleotide transhydrogenase (proton pump), alpha subunit (part1)	*pntA*	ACIAD3079	1.31	1.34	6.03	1.84
Ketol-acid reductoisomerase	*ilvC*	ACIAD3102	4.21	2.38	2.07	2.78
Malate dehydrogenase	*mdh*	ACIAD3155	1.77	1.49	2.65	1.8
Alcohol dehydrogenase	*adhA*	ACIAD3339	6.73	18.54	10	7.73
Fatty acyl-CoA reductase	*acr1*	ACIAD3383	1.47	2.68	1.87	2.29
ssDNA exonuclease, 5′– > 3′ specific, Mg dependent	*recJ*	ACIAD3500	1.61	1.32	1.6	1.46
Pyruvate dehydrogenase (acetyl-transferring), homodimeric type	*aceE*	ACIAD3507	1.49	1.96	1.89	1.66
NADH dehydrogenase II	*ndh*	ACIAD3633	1.84	2.25	4.33	2.75
**GTP binding**						
Peptide chain release factor 3	*prfC*	ACIAD3095	1.58	2.7	2.38	1.32
**Hydratase**						
Bifunctional aconitate hydratase 2/2-methylisocitrate dehydratase	*acnB*	ACIAD2395	5.35	1.69	3.31	1.47
**Kinase**						
Acetate kinase (propionate kinase)	*ack*	ACIAD0541	1.84	4.02	3.53	2.07
Glycerol kinase	*glpK*	ACIAD0930	2.33	3.63	3.53	1.39
Phosphoglycerate kinase	*pgk*	ACIAD1927	1.94	2.05	4.17	1.91
Sulfate ABC transporter periplasmic substrate-binding protein	*cysP*	ACIAD2591	5.4	2.75	4.74	4.29
UDP-*N*-acetylmuramate–L-alanine ligase	*murC*	ACIAD3516	1.47	1.84	1.89	2.11
**Porin activity**						
Porin	*hcaE*	ACIAD1722	3.05	3.37	2.58	2.73
**Down-regulated**						
**Transferase**						
Porphobilinogen deaminase	*hemC*	ACIAD0286	0.69	0.14	0.51	0.63
DNA-directed RNA polymerase subunit beta	*rpoB*	ACIAD0307	0.49	0.72	0.55	0.58
Queuine tRNA-ribosyltransferase	*tgt*	ACIAD0590	0.59	0.74	0.58	0.67
UDP-*N*-acetylglucosamine 1-carboxyvinyltransferase 2	*murA*	ACIAD0660	0.81	0.74	0.8	0.74
Gamma-glutamyltranspeptidase precursor	*ggt*	ACIAD0929	0.34	0.77	0.5	0.41
Bifunctional succinylornithine transaminase/acetylornithine transaminase	*argD*	ACIAD1284	0.4	0.32	0.15	0.26
Arginine *N*-succinyltransferase	*astA*	ACIAD1286	0.48	0.63	0.76	0.63
Succinylglutamate desuccinylase	*astE*	ACIAD1289	0.17	0.82	0.69	0.66
Amidase	*amdA*	ACIAD1618	0.68	0.06	0.58	0.27
Acetyl-CoA acetyltransferase	*dcaF*	ACIAD1689	0.43	0.69	0.54	0.53
Polyphosphate kinase	*ppk*	ACIAD1782	0.77	0.51	0.36	0.48
Tellurium resistance protein	*terZ*	ACIAD1952	0.68	0.65	0.81	0.7
Tellurium resistance protein	*terY*	ACIAD1969	1.91	1.58	1.72	1.75
DNA polymerase III beta	*dnaX*	ACIAD1970	0.78	0.37	0.6	0.73
1-phosphofructokinase	*fruK*	ACIAD1992	0.53	0.69	0.63	0.62
Superoxide dismutase	*sodM*	ACIAD2072	0.7	0.41	0.52	0.61
3-oxoacyl-(acyl carrier protein) synthase III	–	ACIAD2101	0.62	0.67	0.66	0.72
16S rRNA methyltransferase GidB	*gidB*	ACIAD2368	0.59	0.48	0.56	0.52
4-diphosphocytidyl-2C-methyl-D-erythritol kinase	*ipk*	ACIAD2903	0.56	0.35	0.73	0.51
Valyl-tRNA synthetase	*valS*	ACIAD2950	0.81	0.65	0.63	0.69
Xanthine phosphoribosyltransferase	*xpt*	ACIAD3164	0.44	0.52	0.44	0.59
Glycerol-3-phosphate acyltransferase	*plsB*	ACIAD3232	0.6	0.53	0.52	0.69
Guanylate kinase	*gmk*	ACIAD3324	0.6	0.47	0.6	0.63
23S rRNA m(2)G2445 methyltransferase	*rrm*	ACIAD3362	0.5	0.44	0.68	0.44
4-aminobutyrate aminotransferase, PLP-dependent	*gabT*	ACIAD3446	0.1	0.08	0.38	0.19
Histidine phosphatase	*phoR*	ACIAD3558	0.52	0.3	0.59	0.59
Hypoxanthine phosphoribosyltransferase	*hpt*	ACIAD3669	0.47	0.57	0.57	0.51
**DNA RNA-binding**						
GntR family transcriptional regulator	*lldR*	ACIAD0107	0.57	0.6	0.52	0.55
50S ribosomal protein L11	*rplK*	ACIAD0302	0.64	0.63	0.33	0.5
50S ribosomal protein L1	*rplA*	ACIAD0304	0.56	0.69	0.5	0.7
50S ribosomal protein L10	*rplJ*	ACIAD0305	0.74	0.77	0.69	0.61
Translation initiation factor IF-2	*infB*	ACIAD0369	0.48	0.74	0.3	0.69
30S ribosomal protein S15	*rpsO*	ACIAD0401	0.58	0.75	0.5	0.69
Endoribonuclease	*rne*	ACIAD0438	0.55	0.73	0.67	0.74
DNA-binding transcriptional regulator HcaR	*hcaR*	ACIAD0448	0.38	0.49	0.22	0.3
50S ribosomal protein L33	*rpmG*	ACIAD0501	0.45	0.77	0.33	0.4
50S ribosomal protein L28	*rpmB*	ACIAD0502	0.52	0.65	0.39	0.53
30S ribosomal protein S12	*rpsL*	ACIAD0881	0.37	0.49	0.3	0.51
BetI family transcriptional regulator	*betI*	ACIAD1010	0.67	0.69	0.81	0.63
DNA-binding ATP-dependent protease La	*lon*	ACIAD1115	0.55	0.77	0.55	0.48
30S ribosomal protein S21	*rpsU*	ACIAD1331	0.54	0.53	0.44	0.65
Protein RecA	*recA*	ACIAD1385	0.57	0.51	0.59	0.45
30S ribosomal protein S20	*rpsT*	ACIAD1389	0.21	0.35	0.25	0.29
tRNA (uracil-5-)-methyltransferase	*trmA*	ACIAD1645	0.28	0.52	0.41	0.34
Tellurium resistance protein	*terX*	ACIAD1968	0.68	0.65	0.81	0.7
DNA polymerase III beta	*dnaX*	ACIAD1970	0.78	0.37	0.6	0.73
30S ribosomal protein S2	*rpsB*	ACIAD2269	0.55	0.64	0.38	0.53
30S ribosomal protein S1	*rpsA*	ACIAD2347	0.69	0.59	0.36	0.47
Chromosome partitioning protein	*spoOJ*	ACIAD2366	0.56	0.67	0.71	0.81
Chromosome partitioning protein	*soj*	ACIAD2367	0.66	0.45	0.73	0.82
30S ribosomal protein S18	*rpsR*	ACIAD2431	0.52	0.68	0.55	0.49
50S ribosomal protein L9	*rplI*	ACIAD2432	0.55	0.8	0.77	0.61
LysR family transcriptional regulator	*cbl*	ACIAD2597	0.6	0.55	0.74	0.6
50S ribosomal protein L25	*rplY*	ACIAD2908	0.74	0.79	0.65	0.63
50S ribosomal protein L27	*rpmA*	ACIAD2938	0.51	0.69	0.48	0.65
50S ribosomal protein L21	*rplU*	ACIAD2939	0.54	0.64	0.36	0.5
50S ribosomal protein L13	*rplM*	ACIAD3012	0.53	0.6	0.47	0.7
Transcription termination factor Rho	*rho*	ACIAD3038	0.74	0.77	0.6	0.65
50S ribosomal protein L20	*rplT*	ACIAD3046	0.32	0.56	0.44	0.44
50S ribosomal protein L35	*rpmI*	ACIAD3047	0.28	0.42	0.44	0.47
30S ribosomal protein S4	*rpsD*	ACIAD3195	0.44	0.59	0.41	0.58
30S ribosomal protein S11	*rpsK*	ACIAD3196	0.6	0.67	0.51	0.66
30S ribosomal protein S13	*rpsM*	ACIAD3197	0.55	0.48	0.5	0.64
50S ribosomal protein L36	*rpmJ*	ACIAD3198	0.48	0.82	0.61	0.68
50S ribosomal protein L15	*rplO*	ACIAD3200	0.6	0.59	0.47	0.53
50S ribosomal protein L30	*rpmD*	ACIAD3201	0.77	0.73	0.62	0.73
30S ribosomal protein S5	*rpsE*	ACIAD3202	0.51	0.63	0.72	0.76
50S ribosomal protein L18	*rplR*	ACIAD3203	0.45	0.44	0.43	0.53
50S ribosomal protein L6	*rplF*	ACIAD3204	0.44	0.62	0.56	0.63
30S ribosomal protein S8	*rpsH*	ACIAD3205	0.59	0.79	0.44	0.52
30S ribosomal protein S14	*rpsN*	ACIAD3206	0.51	0.58	0.54	0.67
50S ribosomal protein L5	*rplE*	ACIAD3207	0.49	0.75	0.48	0.48
50S ribosomal protein L24	*rplX*	ACIAD3208	0.67	0.63	0.43	0.71
50S ribosomal protein L14	*rplN*	ACIAD3209	0.58	0.54	0.57	0.64
30S ribosomal protein S17	*rpsQ*	ACIAD3210	0.39	0.67	0.3	0.55
50S ribosomal protein L29	*rpmC*	ACIAD3211	0.66	0.53	0.34	0.62
50S ribosomal protein L16	*rplP*	ACIAD3212	0.47	0.45	0.45	0.49
30S ribosomal protein S3	*rpsC*	ACIAD3213	0.56	0.55	0.41	0.61
50S ribosomal protein L22	*rplV*	ACIAD3214	0.62	0.63	0.49	0.59
30S ribosomal protein S19	*rpsS*	ACIAD3215	0.41	0.65	0.36	0.46
50S ribosomal protein L2	*rplB*	ACIAD3216	0.49	0.44	0.43	0.66
50S ribosomal protein L23	*rplW*	ACIAD3217	0.44	0.57	0.42	0.6
50S ribosomal protein L4	*rplD*	ACIAD3218	0.56	0.57	0.48	0.6
30S ribosomal protein S10	*rpsJ*	ACIAD3220	0.71	0.77	0.65	0.7
50S ribosomal protein L19	*rplS*	ACIAD3310	0.32	0.58	0.35	0.4
30S ribosomal protein S16	*rpsP*	ACIAD3313	0.62	0.75	0.58	0.6
50S ribosomal protein L34	*rpmH*	ACIAD3684	0.46	0.61	0.46	0.37
**Synthase**						
Arginyl-tRNA synthetase	*argS*	ACIAD0164	0.58	0.6	0.56	0.69
Aspartyl-tRNA synthetase	*aspS*	ACIAD0609	0.45	0.71	0.39	0.54
Lysyl-tRNA synthetase	*lysS*	ACIAD1069	0.51	0.72	0.61	0.56
Glutamyl-tRNA synthetase	*gltX*	ACIAD3371	0.76	0.67	0.79	0.82
**Activator**						
Transcription termination factor Rho	*rho*	ACIAD3038	0.74	0.77	0.6	0.65
**Ligase**						
GMP synthase	*guaA*	ACIAD0151	0.54	0.39	0.45	0.56
Arginyl-tRNA synthetase	*argS*	ACIAD0164	0.58	0.6	0.56	0.69
Aspartyl-tRNA synthetase	*aspS*	ACIAD0609	0.45	0.71	0.39	0.54
Lysyl-tRNA synthetase	*lysS*	ACIAD1069	0.51	0.72	0.61	0.56
Cyanophycin synthetase	–	ACIAD1279	0.19	0.12	0.23	0.23
Amidase	*amdA*	ACIAD1618	0.68	0.06	0.58	0.27
CTP synthetase	*pyrG*	ACIAD2003	0.82	0.74	0.62	0.7
Bifunctional biotin carboxylase/biotin carboxyl carrier protein	*bccA*	ACIAD2517	0.52	0.48	0.67	0.59
Pantoate–beta-alanine ligase	*panC*	ACIAD3060	0.79	0.82	0.71	0.64
Glutamyl-tRNA synthetase	*gltX*	ACIAD3371	0.76	0.67	0.79	0.82
**Peptidase**						
Aminopeptidase N	*pepN*	ACIAD2008	0.4	0.24	0.56	0.53
**Hydrolase**						
Endoribonuclease	*rne*	ACIAD0438	0.55	0.73	0.67	0.74
ATP-dependent dsDNA exonuclease (suppression of recBC)	*sbcC*	ACIAD0916	0.72	0.82	0.54	0.6
DNA-binding ATP-dependent protease La	*lon*	ACIAD1115	0.55	0.77	0.55	0.48
Phosphate transporter ATP-binding protein	*pstB*	ACIAD1215	0.58	0.3	0.3	0.55
Succinylglutamic semialdehyde dehydrogenase	*astD*	ACIAD1287	0.47	0.51	0.37	0.38
Succinylarginine dihydrolase	*astB*	ACIAD1288	0.33	0.26	0.35	0.2
Uridylate kinase	*pyrH*	ACIAD1372	0.67	0.79	0.81	0.75
DNA polymerase III beta	*dnaX*	ACIAD1970	0.78	0.37	0.6	0.73
Aminopeptidase N	*pepN*	ACIAD2008	0.4	0.24	0.56	0.53
Formyltetrahydrofolate deformylase	*purU*	ACIAD2554	0.62	0.54	0.57	0.59
GTPase	*obgE*	ACIAD2561	0.52	0.6	0.76	0.7
Major intrinsic multiple antibiotic resistance efflux outer membrane protein precursor	*macB*	ACIAD3110	0.59	0.74	0.42	0.75
Oligoribonuclease	*orn*	ACIAD3118	0.55	0.76	0.74	0.69
Oligopeptidase A	*prlC*	ACIAD3182	0.63	0.72	0.76	0.51
Inositol-1-monophosphatase (IMPase) (inositol-1-phosphatase) (I-1-Pase)	*suhB*	ACIAD3246	0.59	0.65	0.52	0.54
DNA/RNA non-specific endonuclease G protein	–	ACIAD3408	0.47	0.42	0.52	0.53
**Protease**						
DNA-binding ATP-dependent protease La	*lon*	ACIAD1115	0.55	0.77	0.55	0.48
Oligopeptidase A	*prlC*	ACIAD3182	0.63	0.72	0.76	0.51
**ATP-binding**						
ATP-dependent protease ATP-binding subunit ClpX	*clpX*	ACIAD0535	0.82	0.47	0.73	0.72
Phosphate starvation-inducible protein (PhoH-like)	*phoL*	ACIAD3159	0.42	0.45	0.62	0.54
**ATPase**						
High affinity Zn ABC transporter ATP-binding protein	*znuB*	ACIAD0174	0.78	0.49	0.74	0.8
ATP-dependent protease ATP-binding subunit ClpX	*clpX*	ACIAD0535	0.82	0.47	0.73	0.72
**Cell cycle**						
Cell division protein	*ftsZ*	ACIAD3511	0.66	0.42	0.54	0.45
**Chaperone**						
Heat shock protein 90	*htpG*	ACIAD0316	0.51	0.51	0.55	0.66
Heat shock protein HSP33	*hslO*	ACIAD0407	0.36	0.72	0.44	0.64
Porin precursor	*quiX*	ACIAD1715	0.17	0.05	0.33	0.46
Chaperonin GroEL	*groEL*	ACIAD2838	0.63	0.29	0.63	0.72
**Isomerase**						
Alanine racemase 2, PLP-binding, catabolic	*dadX*	ACIAD0116	0.55	0.52	0.47	0.33
Fatty acid oxidation complex subunit alpha	*fadB*	ACIAD0335	0.76	0.74	0.7	0.77
Glutamate-1-semialdehyde aminotransferase	*hemL*	ACIAD1201	0.72	0.72	0.6	0.63
Porin precursor	*quiX*	ACIAD1715	0.17	0.05	0.33	0.46
Triphosphoribosyl-dephospho-CoA synthase	*mdcB*	ACIAD1754	0.25	0.17	0.16	0.19
UDP-*N*-acetylenolpyruvoylglucosamine reductase	*murB*	ACIAD1945	0.25	0.82	0.61	0.52
Phosphoglucosamine mutase	*glmM*	ACIAD3502	0.64	0.61	0.77	0.58
Phosphoserine phosphatase	*serB*	ACIAD3567	0.59	0.44	0.65	0.59
**Lyase**						
Fatty acid oxidation complex subunit alpha	*fadB*	ACIAD0335	0.76	0.74	0.7	0.77
Aspartate ammonia-lyase	*aspA*	ACIAD1744	0.02	0.06	0.03	0.03
Fumarate hydratase	*fumC*	ACIAD1890	0.49	0.47	0.56	0.42
Phosphoenolpyruvate carboxylase	*ppc*	ACIAD3627	0.76	0.53	0.46	0.77
**Oxidoreductase**						
Signal peptide	*lspA*	ACIAD0021	0.81	0.47	0.54	0.79
L-lactate dehydrogenase	*lldD*	ACIAD0108	0.39	0.45	0.44	0.57
D-lactate dehydrogenase	*dld*	ACIAD0109	0.55	0.64	0.64	0.58
D-amino acid dehydrogenase small subunit	*dadA*	ACIAD0115	0.11	0.06	0.12	0.16
Fatty acid oxidation complex subunit alpha	*fadB*	ACIAD0335	0.76	0.74	0.7	0.77
Acyl-CoA dehydrogenase	*acdA*	ACIAD0623	0.49	0.81	0.7	0.76
Malate: quinone oxidoreductase	*mqo*	ACIAD1007	0.2	0.56	0.17	0.58
Catechol 1,2-dioxygenase	*catA*	ACIAD1442	0.65	0.65	0.44	0.28
Acyl-CoA dehydrogenase	*dcaA*	ACIAD1693	0.55	0.63	0.6	0.58
Tellurium resistance protein	*terZ*	ACIAD1968	0.68	0.65	0.81	0.7
Pyruvate dehydrogenase (cytochrome)	*poxB*	ACIAD3381	0.52	0.33	0.42	0.62
NADP + -dependent succinate semialdehyde dehydrogenase	*gabD*	ACIAD3445	0.09	0.11	0.33	0.23
**Helicase**						
ATP-dependent RNA helicase	*rhlB*	ACIAD1314	0.56	0.6	0.42	0.52
**Nuclease**						
Endoribonuclease	*rne*	ACIAD0438	0.55	0.73	0.67	0.74
DNA/RNA non-specific endonuclease G protein	*cafA*	ACIAD0830	0.47	0.42	0.52	0.53
ATP-dependent dsDNA exonuclease (suppression of recBC)	*sbcC*	ACIAD0916	0.72	0.82	0.54	0.6
DNA polymerase III beta	*dnaX*	ACIAD1970	0.78	0.37	0.6	0.73
Oligoribonuclease	*orn*	ACIAD3118	0.55	0.76	0.74	0.69
**DNA polymerase**						
DNA polymerase III beta	*dnaX*	ACIAD1970	0.78	0.37	0.6	0.73
**Kinase**						
Polyphosphate kinase	*ppk*	ACIAD1782	0.77	0.51	0.36	0.48
1-phosphofructokinase	*fruK*	ACIAD1992	0.53	0.69	0.63	0.62
Superoxide dismutase	*sodM*	ACIAD2072	0.7	0.41	0.52	0.61
4-diphosphocytidyl-2C-methyl-D-erythritol kinase	*ipk*	ACIAD2903	0.56	0.35	0.73	0.51
Guanylate kinase	*gmk*	ACIAD3324	0.6	0.47	0.6	0.63

For biological processes, most up- or down-regulated proteins were related to metabolite pathways and amino acid synthesis. Phospho-2-dehydro-3-deoxyheptonate aldolase (Aro, biosynthetic process) and 5-methyltetrahydropteroyltrigluta mate-homocysteine methyltransferase (MetE, methionine formation) in all treatments were most up-regulated, showing an average relative abundance of 15.2 ± 10.2 and 9.8 ± 1.6, respectively. In contrast, enzymes responsible for biosynthesis, e.g., L-lactate dehydrogenase (LldD), pyruvate dehydrogenase (cytochrome, PoxB), and malate synthase G (GlcB), were remarkably down-regulated. Additionally, proteins associated with amino acid metabolism varied significantly across different carbon sources, such as alanine (MurC, PanC, and DadX), aspartate (PyrB, GltK, and AspA), and glutamate (ArgJ, MetE, GltK, Gdh, AstE, and HemL). Proteins involved in DNA replication and stress response showed a diverse profiling change. The up-regulated proteins included DNA polymerase Pol III (DnaX), DNA replication regulator (GlnG, BaeR), and Holliday junction DNA helicase (RuvB), whereas DNA replication regulator (LldR, Cbl), two-component regulatory system response regulator (OmpR), and heat shock proteins (HtpG and HslO) were obviously down-regulated. It is worth mentioning that the relative abundance of all identified ribosomal proteins, including twenty-eight 50S ribosomal proteins and eighteen 30S ribosomal proteins, remarkably decreased in comparison with those in LB treatments (Table 2).

### Changes of Proteins Involved in Metabolism and Energy Production

**FIGURE 4:**
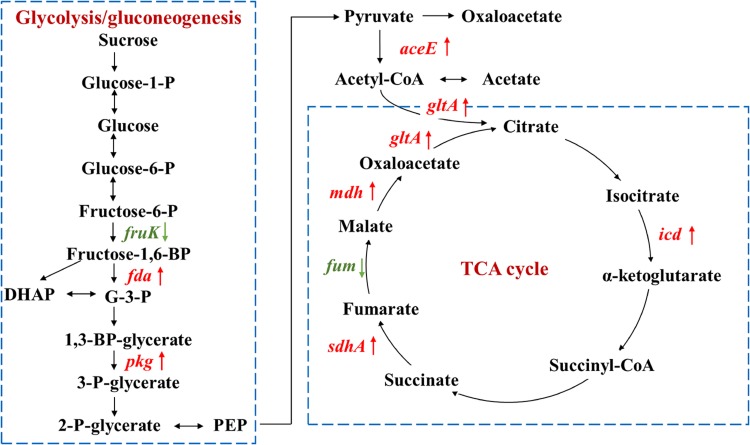
Proteins involved in glycolysis/gluconeogenesis and TCA cycle with significant fold changes in nutrient-deficient media compared to nutrient-rich LB medium. The down- and up-regulated proteins are highlighted in green and red, respectively. Glucose-1-P, glucose 1-phosphate; glucose-6-P, glucose 6-phosphate; fructose-6-P, fructose 6-phosphate; G-3-P, glyceraldehyde 3-phosphate; DHAP, dihydroxyacetone phosphate; 1,3-BP-glycerate, 1,3-bisphospho-D-glycerate; 3-P-glycerate, 3-phospho-D-glycerate; 2-P-glycerate, 2-phospho-D-glycerate; PEP, phosphoenolpyruvate.

As carbon source participates in metabolism and energy production, proteins involved in glycolysis, pyruvate metabolism, and tricarboxylic acid (TCA) cycle were investigated (Figure 4 and Table 2). Except for two enzymes in glycolysis and TCA cycle that were down-regulated, *fruK* and *fum* encoding enzymes for the conversion of fructose-6-P to fructose-1,6-BP and fumarate to malate, other enzymes were all up-regulated, including fructose-1,6-bisphosphate aldolase (Fda), phosphoglycerate kinase (Pgk), pyruvate dehydrogenase (AceE), isocitrate dehydrogenase (Icd), succinate dehydrogenase flavoprotein (SdhA), malate dehydrogenase (Mdh), and citrate synthase (GltA).

### Protein Network Analysis

**FIGURE 5:**
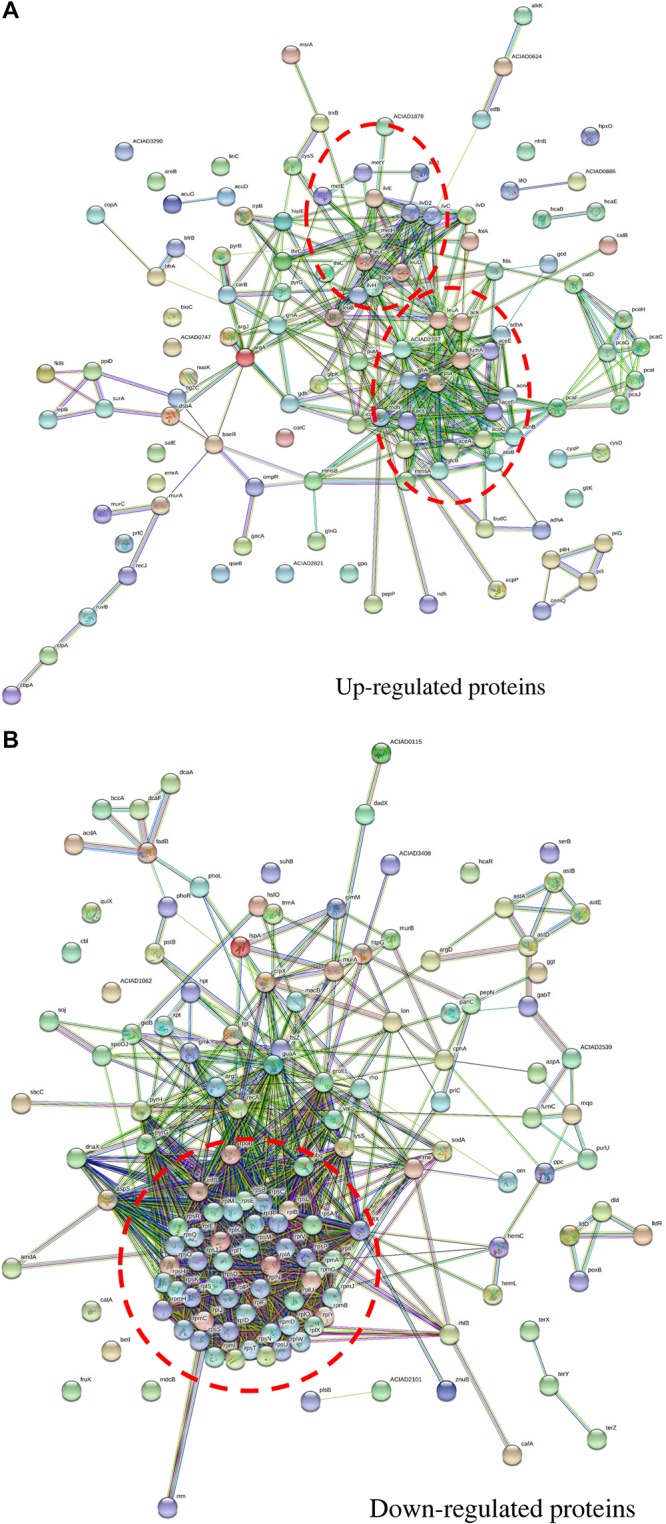
Protein–protein interaction networks of proteins with fold change across carbon sources under mitomycin C-induced DNA damage predicted by STRING. **(A)** The protein–protein interaction network of up-regulated proteins in nutrient-deficient media and core proteins were mainly involved in metabolic pathways. **(B)** The protein–protein interaction network of down-regulated proteins in nutrient-deficient media and core proteins were involved in translation processes. The red circle labels the peptides clustered together in the interaction network with close biological process or similar functions.

The protein–protein interaction network for up-regulated proteins was primarily composed of two clusters (marked by red circles in Figure 5A), both closely related to metabolite processes. Among them, core proteins located in Cluster I were citrate synthase (GltA), malic enzyme (MaeA), methylmalonate-semialdehyde dehydrogenase (MmsA), NADH oxidase (PutA), etc. In Cluster II, core proteins included acetolactate synthase (IlvH), dihydroxy-acid dehydratase (IlvD1), ketol-acid reductoisomerase (IlvC), branched-chain amino acid aminotransferase (IlvE), isopropylmalate isomerase (LeuC and LeuD), bifunctional phosphoribosyl-AMP cyclohydrolase/phosphoribosyl-ATP pyrophosphatase (HisI), etc. In contrast, the protein–protein interaction network for down-regulated proteins had only one cluster (Figure 5B). Proteins related to translation process functioned in this cluster, including ribosomal proteins of *rpl*, *rpm*, and *rps* families.

### Protein Profiles Involved in Stress Response

**FIGURE 6:**
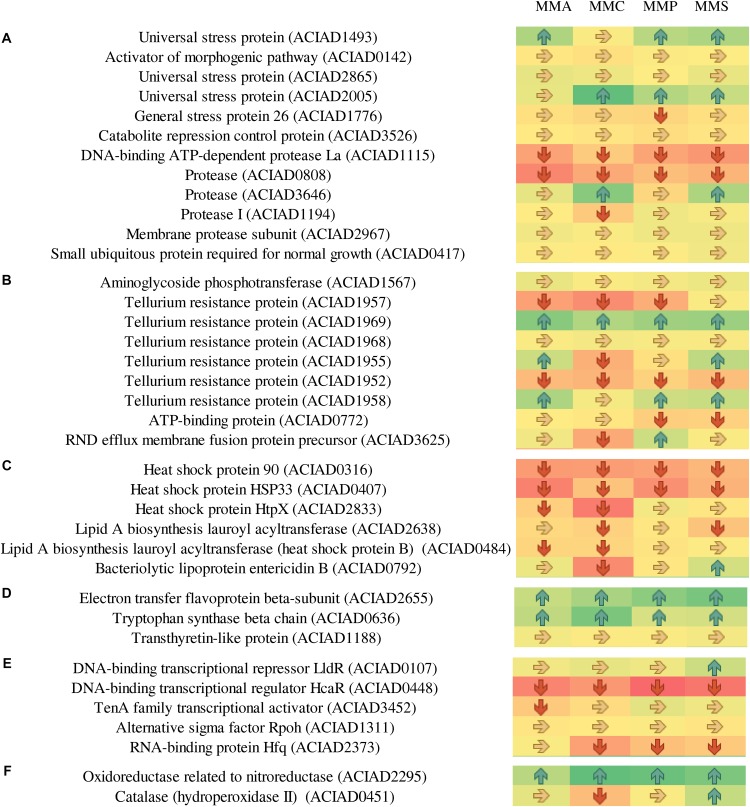
Fold changes of proteins involved in stress response network in relation to different stress response categories. Columns from left to right represent the ratio of proteins of MMA-to-LB, MMC-to-LB, MMP-to-LB, and MMS-to-LB, respectively. **(A)** General stress. **(B)** Antibiotic resistance/detoxification. **(C)** Protein stress. **(D)** Electron transport/transport. **(E)** Activator/repressor. **(F)** Redox stress. **(G)** SOS response/DNA repair. Red arrows refer to significantly down-regulated proteins with relative abundance < 0.83 (*p* < 0.05); green arrows represent significantly up-regulated proteins with relative abundance > 1.20 (*p* < 0.05); yellow arrows refer to proteins with insignificant relative abundance change from 0.83 to 1.20 (*p* > 0.05).

As *A. baylyi* ADP1 cells are commonly used as bioreporter hosts for evaluating genotoxicity in environmental samples, the relative abundance and profiles of stress-related proteins across the four defined media were investigated. Heatmap listed the relative abundance of 80 proteins involved in stress response (Figure 6). It is worth noting that these 80 proteins might show different up- and down-regulation pattern across all four carbon source treatments, differing from those listed in Table 2. These proteins are classified into seven categories according to a previous study (Onnis-Hayden et al., 2009), including 12 general stress-related proteins responsible for keeping steady the biochemical and biophysical homeostasis of cells, 9 antibiotic resistance/detoxification-related proteins responsive to chemical/drug-induced stress or killing, 6 protein stress-related proteins related to oxidative stress, and heat- or chemical-induced protein damage, 3 electron transport/transport-related proteins involved in electron transport, 5 activators/repressors involved in stress response, 2 redox stress-related proteins associated with conditions altering the redox potential of cells, and 43 response/DNA repair-related proteins.

**FIGURE 7:**
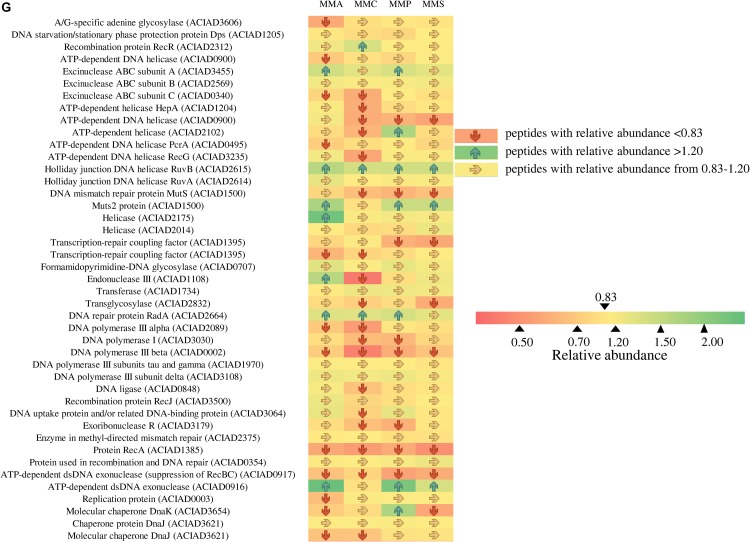
Continued

Among them, only 20 proteins were not influenced by carbon source, including 5 associated with general stress (activator of morphogenic pathway, universal stress protein, catabolite repression control protein, membrane protease subunit, small ubiquitous protein required for normal growth), 2 related to antibiotic resistance/detoxification (aminoglycoside phosphotransferase and tellurium resistance protein), 1 electron transport/transport (transthyretin-like protein), 1 activator/repressor (alternative sigma factor Rpoh), and 12 involved in SOS response/DNA repair [DNA starvation/stationary phase protection protein Dps, excinuclease ABC subunit B, holliday junction DNA helicase (RuvA), helicase, formamidopyrimidine-DNA glycosylase, transferase, DNA polymerase III subunits tau and gamma, DNA polymerase III subunit delta, recombination protein RecJ, enzyme in methyl-directed mismatch repair, protein used in recombination and DNA repair, and chaperone protein DnaJ].

Proteins responsive to protein stress were mainly down-regulated (62.5%) or unchanged (33.3%). In this category, the relative abundance of heat shock protein 90 (0.6 ± 0.1) and heat shock protein HSP33 (0.5 ± 0.2) remarkably decreased in all treatments. Similarly, 40.0% and 55.0% of activators/repressors were down-regulated or unchanged, respectively. The relative abundance of DNA-binding transcriptional regulator HcaR declined to only 0.4 ± 0.1.

In contrast, proteins belonging to electron transporter/transporter were mostly up-regulated, e.g., electron transfer flavoprotein beta-subunit (1.7 ± 0.3), tryptophan synthase beta chain (1.5 ± 0.3), and oxidoreductase related to nitroreductase (2.0 ± 0.3). This was in accordance with the accelerated energy production rates in nutrient-deficient media (Figure 4), as electron transporters/transporters play critical roles in aerobic respiration and serve as the most typical way for bacteria to gain energy (Bjerg et al., 2018; Naradasu et al., 2019).

Across carbon source treatments, profiles of stress-related proteins in MMC treatment behaved significantly different in comparison with others. Nine proteins were down-regulated only in MMC treatment, which are general stress-related protein (Protease I), antibiotic resistance/detoxification-related proteins (tellurium resistance protein and RND efflux membrane fusion protein precursor), SOS response/DNA repair-related proteins (ATP-dependent helicase, endonuclease III, DNA ligase, DNA uptake protein, and/or related DNA-binding protein and rhombosortase), protein stress-related protein (bacteriolytic lipoprotein entericidin B), and redox stress-related catalase (hydroperoxidase II).

Forty-six proteins involved in SOS response/DNA repair were down-regulated in nutrient-deficient treatments (Figure 6G). Among them, three proteins were all down-regulated in all carbon source treatments, including DNA polymerase III subunit beta, protein RecA, and ATP-dependent dsDNA exonuclease (suppression of RecBC). Holliday junction DNA helicase (RuvB) was up-regulated in all nutrient-deficient treatments. The relative abundance of other proteins remained unchanged, e.g., DNA starvation/stationary phase protection protein Dps, excinuclease ABC subunit B, Holliday junction DNA helicase (RuvA), helicase, formamidopyrimidine-DNA glycosylase, transferase, DNA polymerase III subunits tau and gamma, DNA polymerase III subunit delta, recombination protein (RecJ), enzyme in methyl-directed mismatch repair, protein used in recombination and DNA repair, and Chaperone protein (DnaJ).

## Discussion

### Different Behavior of Protein Profiles in Different Carbon Sources

As a model soil microorganism with strong natural transformation competence, *A. baylyi* ADP1 has been fully sequenced (Barbe et al., 2004) and studied for decades (Hare and Gregg-Jolly, 2003; Metzgar et al., 2004; Zheng and Gregg-Jolly, 2004; Buchan and Ornston, 2005). Although *A. baylyi* ADP1 cells have been previously constructed as hosts for DNA damage responsive whole-cell bioreporters in environmental toxicity assessment (Ammerman and Azam, 1987; Barbe et al., 2004; Song et al., 2009; Jiang et al., 2014, 2017; Jia et al., 2016), the DNA damage response network in *A. baylyi* ADP1 is still not well-established. Additionally, previous studies addressing *Acinetobacter*-based bioreporters have demonstrated that carbon sources have significant impacts on their performance. For instance, MMC and MMS were preferred carbon sources to enhance the responsive ratio of ADPWH_recA after DNA damage, whereas MMA and rich medium (LB) did not achieve the optimal performance (Jiang et al., 2015). However, the underlying mechanisms influencing the behavior of ADP1 in response to toxins cultivated with different carbon sources, particularly on translational level, are still not clear and require further study.

From Figures 1, 2 and Supplementary Figure S1, carbon sources significantly affected the protein profiles in *A. baylyi* ADP1 responding to DNA damage by iTRAQ-based proteomics analysis, particularly those involved in metabolism, biosynthesis, transport, energy utilization, and stress response. Previous studies have reported some correlations between carbon source and proteomic profiles. Vandera et al. (2015) conducted a metabolic study on the cultivation of *Arthrobacter phenanthrenivorans* Sphe3 with phenanthrene, phthalate, glucose, or their combinations, suggesting that the influence of aromatic substrates in shaping the protein abundance was related to substrate and amino acid metabolism, as well as stress response. Giardina et al. investigated the influence of glucose starvation and re-feeding on the proteomic profiles of *Saccharomyces cerevisiae*, demonstrating that up-regulated proteins in response to glucose re-feeding included ribosomal subunits and plasma membrane ATPase, whereas those down-regulated included small heat shock proteins, mitochondrial proteins, and gluconeogenic enzymes (Licausi et al., 2013).

Particularly, across different carbon sources, the protein profiles in MMA, MMP, and MMS treatments were similar, all behaving different with those in MMC treatment (Table 2 and Figure 1). To be more precise, more proteins associated with DNA replication and stress were down-regulated in MMC treatment (Figures 2, 6). It might be explained by the ubiquitous roles of citrate as a key cellular intermediate readily assimilated through the central metabolic pathway. This possibly explains the previous observation in Jiang’s work that ADPWH_recA bioreporter cells had the best performance in responding to genotoxins in MMC treatment (Jiang et al., 2015).

The citrate-related proteomic profiles were also previously reported. By exploring the proteome of *Pseudomonas putida* F1, Mandalakis et al. (2013) found the enhancement of the Na^+^/H^+^ antiporter and carbonic anhydrase in citrate treatments compared to benzoate treatments, suggesting that citrate poses more challenges in maintaining pH homeostasis. Our work, for the first time, addressed the critical roles of citrate in shaping the DNA damage responsive proteomic profiles in *A. baylyi* ADP1.

As it is a key technique for exploring gene changes at the level of translation, proteomics has always been applied to reveal the responses of microbes to environmental stresses (McNair et al., 2007; Xu et al., 2007; Chuang et al., 2010; Tian et al., 2013). Transcriptome is another widely used technique in the investigation of microbial responses under stress, which is useful for identifying novel transcripts and analyzing gene expression (Jonas et al., 2007; Sun et al., 2015). The transcriptome of *A. baylyi* ADP1 in response to DNA damage was also explored. Hare et al. applied RNA-Seq to evaluate the DNA damage transcriptome in *A. baylyi* ADP1 induced by mitomycin C with MMS as the medium. Although 2% of genes (66) in the genome of *A. baylyi* ADP1 were inducible by DNA damage, only a few of them were differentially regulated, and most of them were repressed or absent (Hare et al., 2014). To be more precise, 38.4% of all genes in *A. baylyi* ADP1 were repressed in case of DNA damage induced by mitomycin C (Hare et al., 2014). Another study by Aranda et al. (2013) applied DNA microarray to investigate the transcriptional response of *Acinetobacter baumannii* to mitomycin C, confirming the roles of UmuDAb as a direct regulator of DNA damage response instead of RecA. Other studies addressed the transciptomic changes in *A. baylyi* postexposure to environmental stresses, such as low temperature (Ma et al., 2019), pesticides (Pi et al., 2017), and antibiotics (Heo et al., 2014). However, many studies have suggested a weak correlation or discrimination between the transcriptomic and proteomic changes (Barker et al., 2012; Lackner et al., 2012; Su et al., 2016; Fan et al., 2017; Bathke et al., 2019), which is possibly due to the key role that post-transcriptional processes play in the adaptation to stresses (Fan et al., 2017). Therefore, the investigation on the proteomic profiles of *A. baylyi* ADP1 responsive to DNA damage in different carbon sources provides significant molecular information and clue for further interpretation of the mechanism of DNA damage response in *A. baylyi* ADP1.

### Mechanism of Carbon-Dependent Protein Profiles in *A. baylyi* ADP1

The present work addressed the different protein profiles in rich (LB) and nutrient-deficient media (MMA, MMC, MMP, and MMS), possibly explained by carbon catabolite repression (CCR). CCR was first reported in *E. coli* and defined as the repression of pathways or enzyme activities related to the use of secondary carbon sources in the presence of a preferred carbon source, which is an important mechanism allowing efficient carbon source utilization (Ishizuka et al., 1993; Gosset et al., 2004; Görke and Stülke, 2008). A recent study demonstrated that driven by CCR, *E. coli* showed time-series protein profile changes and 96 proteins were remarkably affected, including those responsible for amino acid biosynthesis, cell division and DNA replication, translation, transcription, and central carbon metabolism (Borirak et al., 2015).

For *A. baylyi* ADP1, Huang et al. found that some components in LB medium, e.g., yeast extract, aspartic acid, and asparagine, repressed the expression of *Pu* promoter, explained by CCR (Huang et al., 2008). In addition, CCR is previously reported in *A. baylyi* during the polycyclic aromatic hydrocarbon (PAH) degradation process, in which acetate and succinate exhibited the repression effect and catabolite repression control (Crc) protein was identified (Fischer et al., 2008; Bernard and Habash, 2009; Zimmermann et al., 2009). Accordingly, CCR could potentially influence the performance of *Acinetobacter*-based bioreporters owing to the down-regulated proteins related to adverse conditions in nutrient-deficient media.

### Protein Profiles Related to Translation

It is worth pointing out that almost all translation-related proteins were down-regulated in nutrient-deficient treatments comparing to LB medium (Figure 3). This was consistent with the location of ribosomal proteins in the center of protein–protein interaction network for down-regulated proteins (Figure 5). Ribosomal proteins are reported to conjunct with rRNA and make up the ribosomal subunits involved in the cellular process of translation (Rodnina and Wintermeyer, 2011; Rainer Nikolay et al., 2015), and therefore are positively related to the protein synthesis. Our results indicated that the translation process in *A. baylyi* ADP1 was remarkably inhibited in nutrient-deficient medium, while ribosomal and translation-related proteins were up-regulated, resulting in increased protein synthesis and nutrient uptake in nutrient-rich medium, e.g., LB.

Similar to our findings, previous studies also reported that translation-related proteins were significantly affected by carbon sources. However, no unique conclusion has been drawn whether the translation was promoted by nutritional downshift from nutrient-rich to nutrient-deficient medium. For instance, proteomic analysis deciphering the tacrolimus-overproduction mechanism of *Streptomyces tsukubaensis* revealed that soybean oil addition tuned the pathways of transcriptional regulation, translation, and stress response (Wang et al., 2017). Giardina et al. (2012) studied the proteomic changes in *S. cerevisiae* after transferring from glucose depletion to glucose-rich medium, and the proteins involved in translation including ribosomal subunits and plasma membrane ATPase were up-regulated in glucose-rich medium. A recent iTRAQ-based proteomic study investigated the adaptive strategies of *Rubrivivax benzoatilyticus* JA2 to glucose and demonstrated the down-regulation of proteins involved in DNA replication, translation, electron transport, and photosynthetic machinery (Gupta et al., 2019). These findings suggested that different carbon sources resulted in significant fold changes of translation-related proteins.

### Protein Profiles Involved in Stress Response

As *A. baylyi* ADP1 is commonly used as a bioreporter for genotoxicity assessment, the patterns of DNA damage responsive proteins were comprehensively analyzed. There were three down-regulated proteins (subunit β of DNA polymerase III, RecA, and ATP-dependent dsDNA exonuclease) and one up-regulated protein (Holliday junction DNA helicase RuvB) among all the proteins related to DNA damage response (Figure 6G).

Among the down-regulated proteins, DNA polymerase III is a primary enzyme complex involved in prokaryotic DNA replication, with proofreading capabilities to correct replication mistakes by means of exonuclease activity working at 3′ to 5′ (Kelman and O’Donnell, 1995). The subunit β of DNA polymerase III acts as sliding DNA clamps, keeping the polymerase bound to the DNA (Leshinsky, 2008). RecA is a highly conserved protein in prokaryotic organisms, and its role in SOS response in *E. coli* is binding to single-strand DNA and helping recombinational repair and cleavage of LexA (Michel, 2005; Schlacher et al., 2006; Chen et al., 2008). Although evidence has suggested that RecA is also involved in DNA repair in *Acinetobacter*, possibly responsible for the cleavage of LexA-like UmuDAb (Aranda et al., 2011; DeBruyn et al., 2012), its mechanisms in *Acinetobacter* are not clear. ATP-dependent dsDNA exonuclease participates in DNA replication process when forming the blockage of replication forks. The only up-regulated protein Holliday junction DNA helicase (RuvB) involves the removal of Holliday junction, created by the annealing of newly synthesized strands, from the end of DNA double strands (Fainstein, 2005).

As most proteins (55.3%) involved in SOS response/DNA repair were down-regulated (Figure 6G) in nutrient-deficient media, less DNA damage was repaired and therefore led to a higher induction ratio of ADPWH_recA to genotoxins. Thus, our results provided proteomic explanation that LB was not an ideal medium for genotoxicity assessment in *Acinetobacter*-based bioreporters (Jiang et al., 2015), offering in-depth clues on the roles of carbon sources in post-transcriptional regulation during DNA damage response in *A. baylyi* ADP1.

## Conclusion

In this work, the impacts of carbon source on the proteomic profiles of *A. baylyi* ADP1 responding to DNA damage response were comprehensively explored and discussed by iTRAQ for the first time. Our results unraveled the significant difference in proteomic patterns between nutrient-rich and nutrient-deficient media. Transferase, hydrolase, oxidoreductase, and DNA/RNA-binding proteins were significantly affected by carbon sources, and they were mainly involved in the biological processes of metabolite pathways and amino acid synthesis. Particularly, 80 proteins involved in stress response were significantly altered, hinting the strong influence of carbon source on microbial response to DNA damage and other stresses. These findings provide important mechanistic insights into the adaptation of *A. baylyi* ADP1 to DNA damage stress under nutrient-deficient conditions, suggesting that nutrient-deficient medium, instead of LB, was ideal for genotoxicity assessment in *Acinetobacter*-based bioreporters. Our findings also offer technical tools and theoretical clues to unravel the SOS response network and influential factors in other model microorganisms.

## Data Availability Statement

The datasets generated for this study can be found in the http://www.ncbi.nlm.nih.gov/bioproject/549090.

## Author Contributions

BJ and DZ contributed to the conception and design of the study. BJ, NZ, LL, and GS did the lab work. DZ, YX, GL, and BJ performed the data analysis. DZ, BJ, NZ, and LL performed the statistical analysis. BJ wrote the first draft of the manuscript. DZ, GL, and YX revised the manuscript.

## Conflict of Interest

The authors declare that the research was conducted in the absence of any commercial or financial relationships that could be construed as a potential conflict of interest.

## Footnotes

1 www.uniprot.org

2 https://db.psort.org/

3 https://string-db.org/
