# Supplementary material for: iTRAQ-Based Comparative Proteomic Analysis of Acinetobacter baylyi ADP1 Under DNA Damage in Relation to Different Carbon Sources
Source: Front Microbiol. 2020 Jan 14;10:2906. doi: 10.3389/fmicb.2019.02906 (PMC6971185; doi:10.3389/fmicb.2019.02906)
Supplement: Supplementary file 1 [file Data_Sheet_1.pdf]

Supplementary Material

Supplementary Material

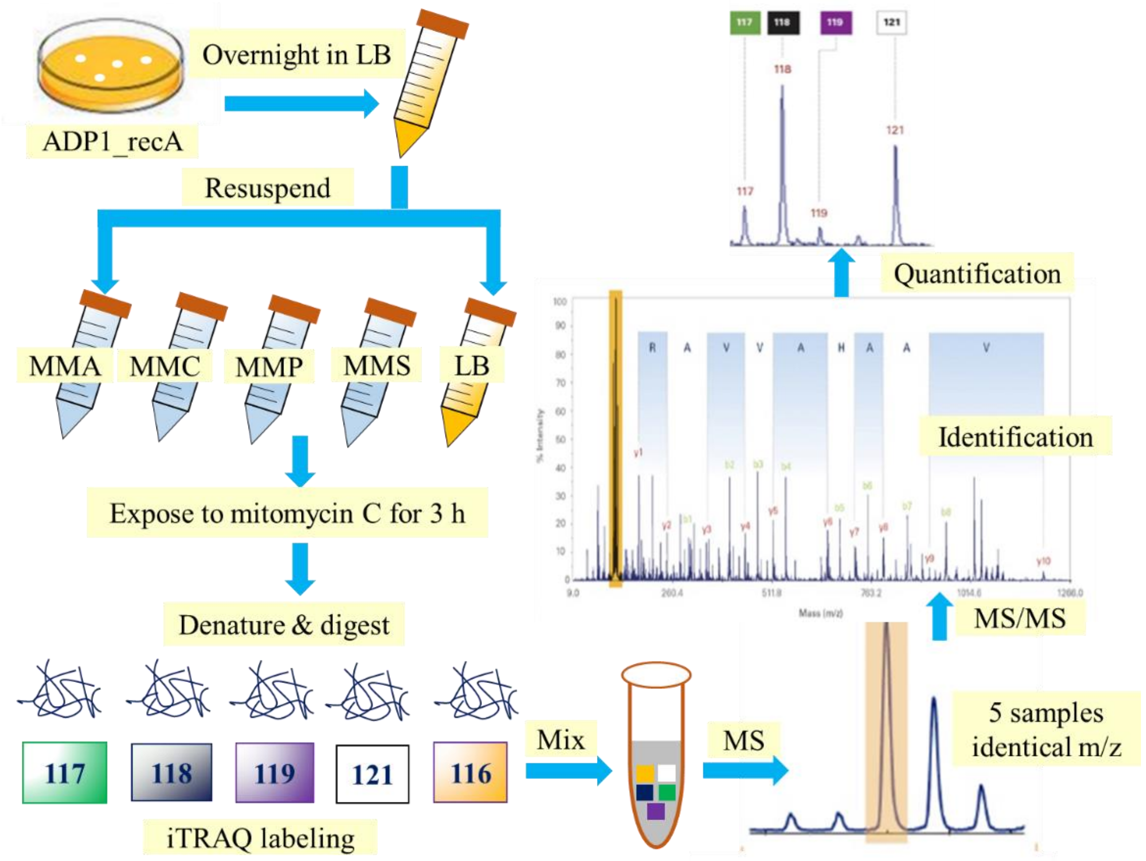

Figure S1. Scheme of exposure, treatment and proteomics analysis. Resuspended cells of *Acinetobacter baylyi* ADP1 were exposed to 1 μM mitomycin C for 3 h and harvested for protein extraction. Samples were processed, digested with trypsin, and the resulting peptides were labeled with 116 (LB), 117 (MMA), 118 (MMC), 119 (MMP) and 121 (MMS). LC/MS/MS was used for the qualitative and quantitative analysis of identified proteins.

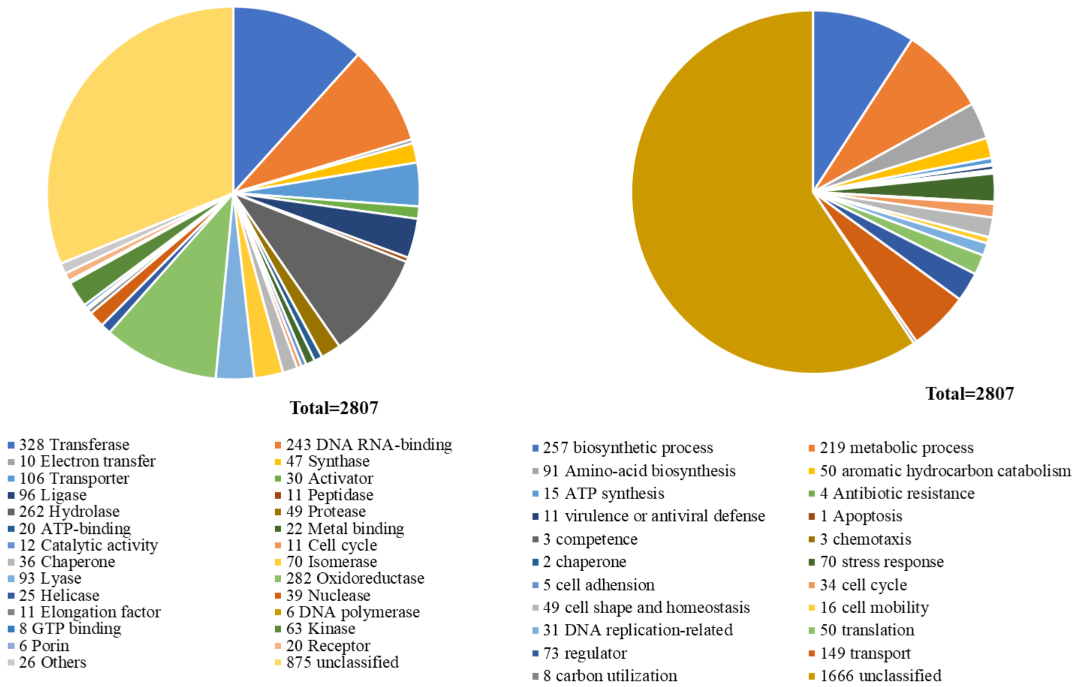

Classification based on molecular function (A)

Classification based on biological process (B)

Figure S2. Classified categories of identified proteins in *A. baylyi* ADP1 by iTRAQ. (A) Classification based on molecular function. (B) Classification based on biological processes. Protein categories with numbers less than 10 are summed up as ‘others’.
